# Anidulafungin is a useful surrogate marker for predicting *in vitro* susceptibility to rezafungin among five *Candida* species using CLSI methods and interpretive criteria

**DOI:** 10.1128/jcm.01129-24

**Published:** 2025-01-22

**Authors:** Marisa L. Winkler, Lalitagauri Deshpande, John H. Kimbrough, Maura Karr, Paul Rhomberg, Abby L. Klauer, Mariana Castanheira

**Affiliations:** 1 Element Iowa City (JMI Laboratories) 138461 , North Liberty, Iowa, USA; University of Utah, Salt Lake City, Utah, USA

**Keywords:** novel agents, antifungal, antifungal susceptibility testing, surrogate testing

## Abstract

This study addresses the use of other echinocandins as surrogate markers to predict the susceptibility of rezafungin against the six most common *Candida* spp. The Clinical Laboratory Standards Institute (CLSI) reference broth microdilution method was performed to test 5,720 clinical isolates of six different *Candida* species. Species-specific interpretative criteria by CLSI breakpoints or epidemiological cutoff values were applied. Essential agreement was 100% within two doubling dilutions for all species and comparisons. The categorical agreement of rezafungin using anidulafungin against all *Candida* spp. was 97.6% (2.9% very major errors [VMEs], 0.2% major errors [MEs], and 2.2% minor errors [miEs]); for caspofungin, it was 99.6% (11.4% VME, 0.09% ME, and 0.19% miE); and for micafungin, it was 99.6% (14.3% VME, 0.15% ME, and 0.17% miE). There were species-specific differences that led to unacceptably high VME for *Candida dubliniensis* with all agents and for *Candida parapsilosis* when caspofungin or micafungin but not anidulafungin was used as the comparator. Genetic analysis showed rezafungin nonsusceptibility correlated well with FKS hotspot mutations. The best-performing surrogate was anidulafungin, which can be used to predict rezafungin susceptible or nonsusceptible in *Candida albicans, Candida glabrata, Candida parapsilosis, Candida tropicalis,* and *Candida krusei* with low error rates and ≥90% essential and categorical agreement. Micafungin or caspofungin can also be used as a surrogate marker for predicting rezafungin susceptible or nonsusceptible in *C. albicans*, *C. glabrata*, *C. tropicalis*, and *C. krusei*. No surrogate performs appropriately to determine rezafungin susceptibility for *C. dubliniensis*.

## INTRODUCTION

Rezafungin is a second-generation echinocandin that has shown activity against a range of *Candida* species including wild-type and azole- and echinocandin-resistant isolates (1–3). Rezafungin has improved chemical and biological properties that are distinct from other echinocandins. Most notably, the modified backbone of rezafungin leads to a stable chemical structure with reduced metabolism and degradation relative to other echinocandins. This results in a longer half-life, which allows for weekly dosing, improved tissue penetration, and reduced organ toxicity (4, 5). Less frequent dosing also results in high front-loaded exposure, which, along with good distribution to and penetration at infection sites may allow earlier mycological clearance from sites of infection with the potential to prevent the emergence of resistance (4, 6–10). Rezafungin was approved in 2023 by the United States Food and Drug Administration for the treatment of candidemia and invasive candidiasis based on clinical efficacy and safety data from the randomized Phase 2 STRIVE and Phase 3 ReSTORE clinical trials (10–12).

The IVD YeastOne AST Plate from ThermoFisher Scientific on the Sensititre platform can be used for rezafungin susceptibility testing (13). However, verification is required to introduce new platforms such as this into the lab. Given the current shortages of laboratory staff and the additional work required to introduce new tests, the use of a surrogate marker to determine rezafungin susceptibility is desirable. The Clinical Laboratory Standards Institute (CLSI) has approved rezafungin susceptible-only breakpoints for *Candida albicans*, *Candida auris*, *Candida glabrata*, *Candida dubliniensis, Candida krusei*, *Candida parapsilosis,* and *Candida tropicalis,* but the FDA only recognizes the *C. parapsilosis* M27M44S breakpoint and either has different breakpoints or does not recognize the breakpoint for other organisms (14). A lack of FDA recognition of breakpoints makes it more difficult for device manufacturers or laboratories to validate testing. Rezafungin disks are commercially available from Hardy Diagnostics with breakpoints for *C. albicans*, *C. glabrata*, *C. parapsilosis*, and *C. tropicalis*, but disk diffusion testing is laborious and more prone to human error than automated antimicrobial susceptibility testing methods. Additionally, performing susceptibility testing of rezafungin can be difficult due to nonspecific drug binding to plates, pipettes, and reservoirs; these problems initially prevented EUCAST breakpoint determination due to intra-laboratory variability (15). Surfactants were tested in broth microdilution plates for rezafungin susceptibility testing, and the use of Tween improved rezafungin test variability (15). Thus, the use of an echinocandin surrogate marker for rezafungin susceptibility is desirable.

Echinocandins are recommended as first-line treatment for serious candidal infections; however, resistance to these agents is rising as these drugs are increasingly used (16–19). Resistance to echinocandins is often mediated by substitutions in hotspot (HS) areas of the *fks* genes in *Candida* spp. (20–24). Therefore, it is important to understand mutations in these genes in isolates that are resistant to one or more echinocandins. To this end, in addition to performing a susceptibility testing comparison using the various echinocandins to identify an adequate surrogate marker for rezafungin, we performed a genetic analysis of isolates with rezafungin MICs above the CLSI-defined breakpoints. This enables us to better understand *fks* mutations that contribute to resistance to rezafungin as well as cross-resistance among other echinocandins.

Given the preferable properties of rezafungin compared to other echinocandins that allow patients to receive once-weekly treatment, being able to easily determine susceptibility to rezafungin for invasive candidal infections is desirable. Therefore, we assessed the ability of anidulafungin, caspofungin, and micafungin to serve as surrogates to predict rezafungin susceptibility using the reference broth microdilution method according to CLSI guidelines. We analyzed a large multiyear collection of the six most common *Candida* spp. causing invasive candidal infections to determine the ability of anidulafungin, caspofungin, or micafungin susceptibility testing to predict rezafungin susceptibility. This study also provides data on cross-resistance between rezafungin and other echinocandins and *fks* mutation analysis among isolates with nonsusceptible rezafungin susceptibility test results.

## MATERIALS AND METHODS

### Organisms

A total of 5,720 isolates of *Candida albicans*, *C. glabrata*, *C. parapsilosis*, *C. tropicalis*, *C. krusei*, and *C. dubliniensis* obtained from 2014 to 2022 in 72 medical centers worldwide were analyzed. These medical centers included 24 in North America (all US census regions), 26 in Europe, 12 in the Asia-Pacific region, and 10 in Latin America. Isolates included 2,603 *C*. *albicans*, 1,156 *C*. *glabrata*, 979 *C*. *parapsilosis*, 598 *C*. *tropicalis*, 194 *C*. *dubliniensis*, and 190 *C*. *krusei*. We tested one isolate per patient, and all isolates were obtained from blood or other normally sterile body sites. Isolates were assessed for purity with the use of BBL CHROMagar Candida (Becton Dickinson and Company, Sparks, MD, USA). All isolates were identified to the species level using matrix-assisted laser desorption ionization time-of-flight mass spectrometry (MALDI-TOF MS; Bruker Daltonics, Billerica, MA, USA). Isolates that did not achieve an acceptable identification by MALDI-TOF MS (score ≥ 2.0) were identified using sequencing-based methods for internal-transcribed region or 28S ribosomal subunit.

### Antifungal susceptibility testing

All isolates were tested by broth microdilution as described in the Clinical and Laboratory Standards Institute document M27 (25). All echinocandins were tested on the same panel with a single inoculum of each organism. The range of antifungal agent concentrations tested was 0.008– 8 mg/L for all tested echinocandins. The MIC result for each agent was recorded after 24 hours of incubation. For all drugs, the MIC was determined as the concentration of agent that caused 50% growth inhibition at 24 hours (14). Breakpoints applied were those defined by CLSI to categorize isolates as susceptible (S), intermediate (I), resistant (R), or nonsusceptible (NS) (14). Epidemiological cutoff values (ECVs) that separate the wild-type (WT) and non-wild-type population (NWT) were used for *C. dubliniensis* and anidulafungin and micafungin, as no breakpoints are defined (26). Table S1 provides these CLSI breakpoints and ECVs. Given the lack of I or R definitions for rezafungin, any MIC result that was not in the S category was defined as NS. Quality control was performed as recommended in CLSI M27M44S and M57S using *C. krusei* ATCC 6258 and *C. parapsilosis* ATCC 22019 (14, 26).

### Analysis of results

All MIC data for rezafungin were compared to micafungin, anidulafungin, and caspofungin using regression statistics and scattergram as previously described (27). Essential agreement was determined at zero doubling dilutions, one doubling dilution, and two doubling dilutions. A very major error (VME) was defined as a false-susceptible error where there was a NS result for rezafungin but a susceptible result for caspofungin, anidulafungin, or micafungin. This was calculated by dividing the number of isolates susceptible to the surrogate antifungal agent by the total number of isolates that tested NS to rezafungin (28). Major error (ME) was defined as a false-resistant error, where rezafungin was defined as susceptible, but anidulafungin, caspofungin, or micafungin was resistant. This was calculated as the number of isolates that tested resistant to the surrogate antifungal by the total number of isolates that tested susceptible to rezafungin. Minor error (miE) was defined as the comparator being intermediate but rezafungin susceptible or NS. The minor error rate was calculated by dividing the number of isolates that tested intermediate to the surrogate antifungal divided by the total number of tested isolates. The goal categorical agreement for rezafungin and the surrogate marker was ≥90% and VME ≤ 3%.

### Echinocandin resistance analysis

All 35 *Candida* spp. isolates with rezafungin MICs above the susceptibility breakpoint were selected for sequencing. For 34 isolates, total genomic DNA was used as input material for library construction prepared using the Nextera XT or Illumina DNA Prep library construction protocol and index kit (Illumina, San Diego, CA, USA) following the manufacturer’s instructions. Sequencing was performed on a MiSeq or NextSeq 1000 Sequencer (Illumina, San Diego, CA, USA). Reads were error corrected using BayesHammer. Each sample was assembled using a reference-guided assembly in DNASTAR SeqMan NGen version 14.0 (Madison, WI, USA). DNA regions encoding *fks* were compared to the sequences available in the literature. One isolate preceded current methods by whole-genome sequencing, and testing for this isolate was performed with singleplex PCR as previously described (29). The sequence from this isolate is unable to be uploaded due to this testing method. The presence or absence of mutations in the hot-spot regions of *FKS1* and *FKS2* for any organisms was determined as previously described (1, 21, 30).

## RESULTS

### Antifungal susceptibility testing and agent comparisons

#### Candida albicans

The rezafungin MIC range for 2,603 *C*. *albicans* isolates tested (Table 1) was ≤0.008–1 mg/L (Table 2). Three *C. albicans* isolates displayed NS MIC values to rezafungin, one was R to anidulafungin, five were R to caspofungin, and five were R to micafungin (Table 2). The EA of rezafungin compared to caspofungin in *C. albicans* at identical MIC values (zero doubling dilutions) was 37.0%, at one doubling dilution it was 84.4%, and at two doubling dilutions it was 100.0% (Table 3). The EA of anidulafungin compared to rezafungin in *C. albicans* at zero doubling dilutions was 50.4%, at one doubling dilution 94.3%, and at two doubling dilutions 100.0% (Table 3). The EA of micafungin compared to rezafungin at zero doubling dilutions was 25.3%, at one doubling dilution 73.2%, and at two doubling dilutions 100.0%. When rezafungin CLSI MICs were compared to anidulafungin CLSI MICs, the results were zero VMEs (0.0%) and two miEs (<0.1%) (Table 4). ME could not be calculated, as there were no intermediate isolates to any tested agents. The findings were the same when rezafungin CLSI breakpoints were compared to caspofungin or micafungin breakpoints: there were zero VME and two (<0.1%) MEs (Table 3).

**TABLE 1:** MIC distributions of rezafungin, anidulafungin, caspofungin, and micafungin versus *Candida* spp.^*a*^^,^^*b*^

Species (no. tested)	Antifungal agent	Number of isolates at each tested MIC with nonsusceptible breakpoint or non-WT by ECV (only for *C. dubliniensis* with anidulafungin and micafungin) bolded											%S/WT
		≤0.008	0.015	0.03	0.06	0.12	0.25	0.5	1	2	4	≥8	
*Candida albicans* (2,603)	RZF	265	806	1,034	434	57	4	**2**	**1**	**0**	**0**	**0**	99.9
	AND	528	923	859	260	29	2	3	**1**	**0**	**0**	**0**	99.9
	CAS	434	1,359	715	85	5	0	0	**2**	**2**	**1**	**0**	99.8
	MCF	835	1,470	288	4	1	0	0	**3**	**2**	**0**	**0**	99.8
*Candida glabrata* (1,156)	RZF	4	40	520	459	93	12	5	**9**	**13**	**1**	**0**	97.6
	AND	1	7	140	643	316	16	**5**	**8**	**11**	**9**	**0**	97.1
	CAS	13	155	670	254	30	9	**4**	**7**	**4**	**4**	**6**	97.8
	MCF	200	734	169	15	9	**7**	**5**	**9**	**3**	**5**	**0**	97.5
*Candida parapsilosis* (979)	RZF	1	1	2	1	3	25	201	478	263	**0**	**4**	99.6
	AND	0	2	2	1	1	5	57	224	590	96	**1**	99.9
	CAS	0	4	1	8	115	563	264	22	2	0	**0**	100.0
	MCF	1	3	1	1	3	5	153	729	83	0	**0**	100.0
*Candida tropicalis* (598)	RZF	47	160	242	120	23	4	**0**	**1**	**1**	**0**	**0**	99.7
	AND	52	185	231	102	22	4	0	**2**	**0**	**0**	**0**	99.7
	CAS	38	264	232	50	10	2	0	**0**	**1**	**0**	**1**	99.7
	MCF	42	210	263	76	5	0	0	**1**	**1**	**0**	**0**	99.7
*Candida dubliniensis* (194)	RZF	5	10	74	74	28	**1**	**2**	**0**	**0**	**0**	**0**	98.5
	AND	1	17	89	64	21	**0**	**2**	**0**	**0**	**0**	**0**	99.0
	CAS	0	46	122	22	1	1	0	1	1	0	0	ND
	MCF	12	91	84	4	1	**0**	**0**	**2**	**0**	**0**	**0**	99.0
*Candida krusei* (190)	RZF	1	53	102	25	9	0	**0**	**0**	**0**	**0**	**0**	100.0
	AND	0	6	71	95	17	1	0	**0**	**0**	**0**	**0**	100.0
	CAS	0	1	2	78	86	22	1	**0**	**0**	**0**	**0**	100.0
	MCF	0	1	4	93	90	2	0	**0**	**0**	**0**	**0**	100.0

^*a*^ MIC, minimum inhibitory concentration; WT, wild type; ECV, epidemiological cutoff value; S, susceptible; RZF, rezafungin; AND, anidulafungin; CAS, caspofungin; MCF, micafungin; ND, not determined due to lack of ECV or S breakpoint.

^*b*^ Bolded values indicate non-susceptible, resistant, or non-wild-type isolates.

**TABLE 2:** Use of other agents to predict susceptibility patterns of rezafungin using 5,720 isolates of *Candida* spp. from a global surveillance program^*a*^

Species (no. tested)	Rezafungin category	No. (%) in anidulafungin category			No. (%) in caspofungin category			No. (%) in micafungin category		
		S	I	R	S	I	R	S	I	R
*C. albicans* (2,603)	S	2,600 (99.9)	0	0	2,598 (99.9)	0	2 (0.08)	2,598 (99.9)	0	2 (0.08)
	NS	0	2 (0.08)	1 (0.04)	0	0	3 (0.1)	0	0	3 (0.1)
*C. glabrata* (1,156)	S	1,107 (95.8)	16 (1.4)	10 (0.9)	1,122 (97.1)	8 (0.7)	3 (0.2)	1,118 (96.7)	9 (0.8)	6 (0.5)
	NS	0	0	23 (2.0)	0	1 (0.09)	22 (1.9)	0	0	23 (2.0)
*C. parapsilosis* (979)	S	882 (90.1)	93 (9.5)	0	975 (99.6)	0	0	975 (99.6)	0	0
	NS	0	3 (0.3)	1 (0.1)	4 (0.4)	0	0	4 (0.4)	0	0
*C. tropicalis* (598)	S	596 (99.7)	0	0	596 (99.7)	0	0	596 (99.7)	0	0
	NS	0	0	2 (0.3)	0	0	2 (0.3)	0	0	2 (0.3)
*C. dubliniensis* (194)	WT	191 (98.5)	0	0	NA	NA	NA	191 (98.5)	0	0
	I	0	0	0	NA	NA	NA	0	0	0
	NWT	1 (0.05)	0	2 (1.0)	NA	NA	NA	1 (0.05)	0	2 (0.1)
*C. krusei* (190)	S	190 (100.0)	0	0	189 (99.5)	1	0	190 (100.0)	0	0
	NS	0	0	0	0	0	0	0	0	0

^*a*^ MIC or ECV interpretative criteria in references (14, 31); S, susceptible; I, intermediate; R, resistant; NS, no intermediate or resistant breakpoints are defined for rezafungin, so non-susceptible isolates were marked as NS; WT, wild type; and NWT, non-wild type.

**TABLE 3:** Essential agreement of rezafungin and other echinocandins for all tested *Candida* spp. at zero, one, and two doubling dilutions

Organisms/ organism groups (no. of isolates)	No. of isolates within essential agreement (cumulative %)								
	Caspofungin/rezafungin			Anidulafungin/rezafungin			Micafungin/rezafungin		
	0 dilutions	1 dilution	2 dilutions	0 dilutions	1 dilution	2 dilutions	0 dilutions	1 dilution	2 dilutions
*Candida* spp. (5,720)	1,791 (31.3)	2,529 (75.5)	1,400 (100.0)	2,653 (46.4)	2,753 (94.5)	314 (100.0)	1,559 (27.3)	2,565 (72.1)	1,596 (100.0)
*Candida albicans* (2,603)	964 (37.0)	1,232 (84.4)	407 (100.0)	1,312 (50.4)	1,143 (94.3)	148 (100.0)	658 (25.3)	1,248 (73.2)	697 (100.0)
*Candida glabrata* (1,156)	485 (42.0)	561 (90.5)	110 (100.0)	442 (38.2)	660 (95.3)	54 (100.0)	81 (7.0)	461 (46.9)	614 (100.0)
*Candida parapsilosis* (979)	56 (5.7)	262 (32.5)	661 (100.0)	325 (33.2)	574 (91.8)	80 (100.0)	537 (54.9)	404 (96.1)	38 (100.0)
*Candida tropicalis* (598)	216 (36.1)	311 (88.1)	71 (100.0)	373 (62.4)	210 (97.5)	15 (100.0)	231 (38.6)	301 (89.0)	66 (100.0)
*Candida dubliniensis* (194)	59 (30.4)	104 (84.0)	31 (100.0)	137 (70.6)	55 (99.0)	2 (100.0)	29 (14.9)	92 (62.4)	73 (100.0)
*Candida krusei* (190)	11 (5.8)	59 (36.8)	120 (100.0)	64 (33.7)	111 (92.1)	15 (100.0)	23 (12.1)	59 (43.2)	108 (100.0)

**TABLE 4:** Categorical agreement and error rates (%) when other agents were used to predict the rezafungin susceptibility of *Candida* spp.^*e*^

	Anidulafungin				Caspofungin				Micafungin			
Species (no. tested)	CA	VME^*a*^	ME^*b*^	miE^*c*^	CA	VME^*a*^	ME^*b*^	miE^*c*^	CA	VME^*a*^	ME^*b*^	miE^*c*^
*C. albicans* (2,603)	2,601 (99.9%)	0	0	2 (0.08)	2,601 (99.9%)	0	2 (0.08)	0	2,601 (99.9%)	0	2 (0.08)	0
*C. glabrata* (1,156)	1,130 (97.8%)	0	10 (0.9)	16 (1.4)	1,144 (99.0%)	0	3 (0.3)	9 (0.8)	1,141 (98.7%)	0	6 (0.5)	9 (0.8)
*C. parapsilosis* (979)	883 (90.2%)	0	0	96 (9.81)	975 (99.6%)	4 (100)	0	0	975 (99.6%)	4 (100)	0	0
*C. tropicalis* (598)	598 (100.0%)	0	0	0	598 (100.0%)	0	0	0	598 (100.0%)	0	0	0
*C. dubliniensis* (194)	193 (99.5%)	1 (33.3)	0	0	NA	NA	NA	NA	193 (99.5%)	1 (33.3)	0	0
*C. krusei* (190)	190 (100.0%)	0	0	0	189 (99.5%)	0	0	1 (0.53)	190 (100.0%)	0	0	0
All *Candida* spp. (5,720)	5,595 (97.8%)	1 (2.9)	10 (0.2)	114 (2.0)	5,507 (99.7%)^*d*^	4 (11.4)^*d*^	5 (0.09)^*d*^	10 (0.2)	5,698 (99.6%)	5 (14.3)	8 (0.1)	9 (0.2)

^*a*^ VME calculated as the number of isolates susceptible to the surrogate antifungal (anidulafungin, caspofungin, or micafungin) divided by the number of isolates resistant to rezafungin (28).

^*b*^ ME calculated as the number of isolates that tested resistant to the surrogate antifungal divided by the number of isolates susceptible to rezafungin.

^*c*^ MiE calculated as the number of isolates that tested intermediate to the surrogate antifungal divided by the total number of isolates tested.

^*d*^ For caspofungin, there was a total of 5,526 isolates since there were no breakpoints or ECV for *C. dubliniensis.*

^*e*^ CA, categorical agreement; VME, very major error; ME, major error; miE, minor error.

#### Candida glabrata

Rezafungin MICs for 1,156 *C*. *glabrata* ranged from ≤0.002 to 4 mg/L, and 23 tested *C. glabrata* isolates were NS to rezafungin (Table 1). A total of 33 isolates were resistant to anidulafungin, 25 were resistant to caspofungin, and 29 were resistant to micafungin (Table 2). The EA of caspofungin and rezafungin at zero doubling dilutions for *C. glabrata* was 42.0%, at one doubling dilution 90.5%, and at two doubling dilutions 100.0% (Table 3). For anidulafungin and rezafungin, EA at 0 doubling dilutions was 38.2%, at 1 doubling dilution 95.3%, and at 2 doubling dilutions 100.0%. When micafungin and rezafungin MICs were compared, the essential agreement was 7.0% at 0 doubling dilutions, 46.9% at one doubling dilution, and 100.0% at two doubling dilutions. Using anidulafungin as a surrogate for rezafungin, there were 0 VME (0.0%), 10 (0.9%) ME, and 16 (1.4%) miEs (Table 4). With caspofungin as a surrogate, there were zero VME (0.0%), three (0.3%) ME, and nine (0.8%) miEs (Table 4). When micafungin was compared to rezafungin, there were zero VME (0.0%), six (0.5%) ME, and nine (0.8%) miEs (Table 4).

#### Candida parapsilosis

Against the 979 *C*. *parapsilosis* isolates tested, the rezafungin MICs ranged from 0.008 to >2 mg/L (Table 1). Four isolates were NS to rezafungin, one R to anidulafungin, and none were R to caspofungin or micafungin (Table 2). The EA of caspofungin and rezafungin at zero doubling dilutions against *C. parapsilosis* was 5.7%, at one doubling dilution 32.5%, and at two doubling dilutions 100.0%. For anidulafungin and rezafungin, the rate of EA at zero doubling dilutions was 33.2%, at one doubling dilution 91.8%, and at two doubling dilutions 100.0%. At zero doubling dilutions, the EA of micafungin and rezafungin was 54.9%, at one doubling dilution 96.1%, and at two doubling dilutions 100.0%. When anidulafungin was compared to rezafungin for *C. parapsilosis* (Table 4), the VME was 0 (0.0%), ME 0 (0.0%), and miE 96 (9.8%). Comparison of caspofungin or micafungin to rezafungin led to four VME (100%), zero ME (0.0%), and zero miE (0.0%; Table 4).

#### Candida tropicalis

The rezafungin MIC values for 598 *C*. *tropicalis* isolates tested ranged from ≤0.008 to 2 mg/L (Table 1). Two isolates were NS to rezafungin and two were R to anidulafungin, caspofungin, or micafungin (Table 2). The EA of caspofungin and rezafungin at zero doubling dilutions was 36.1%, at one doubling dilution 88.1%, and at two doubling dilutions 100.0% (Table 3). For anidulafungin and rezafungin, the EA at zero doubling dilutions was 62.4%, at one doubling dilution 97.5%, and at two doubling dilutions 100.0%. The EA of micafungin and rezafungin at zero doubling dilutions was 38.6%, at one doubling dilution 89.0%, and at two doubling dilutions 100.0%. When comparing anidulafungin, caspofungin, or micafungin to rezafungin, there were no VME (0.0%), no ME (0.0%), and no miE (0.0%) (Table 4).

#### Candida dubliniensis

There were 194 *C*. *dubliniensis* isolates tested for echinocandin susceptibility (Table 1). The MICs for rezafungin ranged from ≤0.002 to 0.5 mg/L. Three isolates tested NS to rezafungin, two non-WT to anidulafungin according to ECV (26), and two isolates non-WT to micafungin (Table 2). There are no defined ECV or breakpoints for caspofungin and *C. dubliniensis*, so a categorical agreement comparison was not performed with caspofungin. The EA of caspofungin and rezafungin was 30.4% at zero doubling dilutions, 84.0% at one doubling dilution, and 100.0% at two doubling dilutions (Table 3). Anidulafungin and rezafungin had an EA of 70.6% at zero doubling dilutions, 99.0% at one doubling dilution, and 100.0% at two doubling dilutions for *C. dubliniensis*. The EA of micafungin and rezafungin was 14.9% at zero doubling dilutions, 62.4% at one doubling dilution, and 100.0% at two doubling dilutions. When the anidulafungin ECV was compared to the rezafungin MIC, there was one VME (33.3%), zero ME (0.0%), and zero miE (Table 4). When the micafungin ECV was compared to the rezafungin MIC, there was one VME (33.3%), zero ME (0.0%), and zero miE (Table 4).

#### Candida krusei

A total of 190 *C*. *krusei* isolates were tested (Table 1). MICs for rezafungin ranged from 0.008 to 0.12 mg/L, and no isolates were NS to rezafungin. One isolate was I to caspofungin, but no isolates tested R to anidulafungin, caspofungin, or micafungin (Table 2). The EA of caspofungin and rezafungin MIC testing for *C. krusei* at zero doubling dilutions was 5.8%, at one doubling dilution 36.8%, and at two doubling dilutions 100.0%. The anidulafungin and rezafungin EA was 33.7% at zero doubling dilutions, 92.1% at one doubling dilution, and 100.0% at two doubling dilutions (Table 3). Micafungin and rezafungin EA was 12.1% at zero doubling dilutions, 43.2% at one doubling dilution, and 100.0% at two doubling dilutions. When comparing anidulafungin or micafungin to rezafungin (Table 4) there were zero VME, zero ME, and zero miE (0.0%; Table 4). For caspofungin compared to rezafungin, there were zero VME (0.0%), zero ME (0.0%), and one miE (0.5%; Table 4).

### Genetic analysis

A total of 35 rezafungin-NS isolates had *fks1* and *fks2* sequencing performed. The NS isolates were predominantly from North America (20/35, 57.1%), but there were three rezafungin-NS isolates from South America (3/35, 8.6%) and 12 isolates from Europe (12/35, 34.3%). There were no NS isolates represented in surveillance from the Asia-Pacific region. Analysis of rezafungin-NS isolates included 3 *C*. *albicans*, 23 *C*. *glabrata*, 2 *C*. *tropicalis*, 4 *C*. *parapsilosis*, and 3 *C*. *dubliniensis* isolates (Table 5).

**TABLE 5:** Rezafungin-nonsusceptible *Candida* spp. with genetic analysis of FKS1 and FKS2 proteins for hotspot (HS) mutations^*d*^^,^^*e*^

Organism	MIC (mg/L)				FKS1 HS1	FKS1 HS2	FKS2 HS1	FKS2 HS2
	Rezafungin	Anidulafungin	Caspofungin	Micafungin				
*Candida dubliniensis*	0.25	0.12	0.25	0.12	Wild type	Wild type	NT	NT
*Candida dubliniensis*	0.5	0.5	1	1	S645P	Wild type	NT	NT
*Candida dubliniensis*	0.5	0.5	2	1	S645P	Wild type	NT	NT
*Candida albicans*	1	0.5	4	2	S645P	Wild type	NT	NT
*Candida albicans*	0.5	1	2	2	S645P	Wild type	NT	NT
*Candida albicans*	0.5	0.5	2	1	S645P	Wild type	NT	NT
*Candida tropicalis*	2	1	>8	2	S654P	Wild type	NT	NT
*Candida tropicalis*	1	1	2	1	F650S	Wild type	NT	NT
*Candida glabrata*	2	2	1	1	Wild type	Wild type	S663P	Wild type
*Candida glabrata*	1	2	0.5	0.5	Wild type	Wild type	S663P	Wild type
*Candida glabrata*	1	1	0.25	0.25	Wild type	Wild type	F659del	Wild type
*Candida glabrata*	2	4	2	0.5	Wild type	Wild type	S663P	Wild type
*Candida glabrata*	1	1	1	0.5	Wild type	Wild type	F659del	Wild type
*Candida glabrata*	2	2	1	1	Wild type	Wild type	S663P	Wild type
*Candida glabrata*	2	4	4	4	Disrupted^*a*^	Disrupted^*a*^	S663P	Wild type
*Candida glabrata*	2	2	>4	1	Wild type	Wild type	S663P	Wild type
*Candida glabrata*	2	4	1	0.5	Wild type	Wild type	S663P	Wild type
*Candida glabrata*	2	4	1	0.25	Wild type	Wild type	S663P	Wild type
*Candida glabrata*	1	2	4	1	S629P	Wild type	Wild type	Wild type
*Candida glabrata*	2	4	>4	4	S629P	Wild type	Wild type	Wild type
*Candida glabrata*	1	2	1	1	Wild type	Wild type	S663P	Wild type
*Candida glabrata*	1	2	2	0.5	S629P	Wild type	Wild type	Wild type
*Candida glabrata*	2	2	4	1	Wild type	Wild type	S663P	Wild type
*Candida glabrata*	1	4	4	1	S629P	Wild type	Wild type	Wild type
*Candida glabrata*	1	2	2	2	Wild type	Wild type	S663P	Wild type
*Candida glabrata*	2	2	8	2	Wild type	Wild type	S663P	Wild type
*Candida glabrata*	2	2	2	2	Wild type	Wild type	S663P	Wild type
*Candida glabrata*	4	4	>8	4	S629P	Wild type	S663P	Wild type
*Candida glabrata*	2	4	>8	4	S629P	Wild type	S663P	Wild type
*Candida glabrata*	1	1	1	0.25	F625S	Wild type	Disrupted^*b*^	Disrupted^*b*^
*Candida glabrata*	2	4	>4	4	S629P^*c*^	Wild type	S663P	Wild type
*Candida parapsilosis*	4	4	1	1	Wild type	Wild type	NT	NT
*Candida parapsilosis*	4	4	1	2	Wild type	Wild type	NT	NT
*Candida parapsilosis*	>2	>4	0.12	1	Wild type	Wild type	NT	NT
*Candida parapsilosis*	4	4	2	1	Wild type	Wild type	NT	NT

^*a*^ Isolate’s *fks1* bore an 8-bp insertion producing a frameshift mutation at F596 with a nonsense mutation introduced at S629.

^*b*^ Isolate’s *fks2* bore a 1-bp insertion producing a frameshift mutation at E573 with a nonsense mutation introduced at S617.

^*c*^ Isolate’s *fks1* possessed a putative recombination event incorporating nucleotide 785–3,353 from *fks*2 in the region of *fks1* between 683 and 3,252 bp. The S629P alteration is given with respect to the location within FKS1 and corresponds to S663P in FKS2. Additional alterations between FKS1 and FKS2 resultant of the putative recombination event outside the FKS1 HS1 have been omitted.

^*d*^ MIC, minimum inhibitory concentration; NT, not tested.

^*e*^ Data available at https://www.ncbi.nlm.nih.gov/bioproject/1199623.

The identified mutations in FKS1 and FKS2 hotspots 1 (HS1) and 2 (HS2) varied by species (Table 5). All rezafungin-NS *C. albicans* isolates had S645P substitutions in the FKS1 HS1. These isolates were all pan-NS to other echinocandins. The rezafungin-NS *C. glabrata* isolates had mutations in the FKS1 HS1, FKS1 HS2, and FKS2 HS2. Among the 23 rezafungin-NS *C. glabrata* isolates, 22 (95.7%) were pan-echinocandin R, and the remaining isolate was caspofungin-I while R against anidulafungin and micafungin. One *C. dubliniensis* lacked FKS1 or FKS2 HS mutations and exhibited a rezafungin MIC of 0.25 mg/L, while the other two isolates with MICs of 0.5 mg/L had S645P mutations in FKS1 HS1. The isolate without identified FKS1 or FKS2 HS1 mutations was at the upper end of the WT population ranges for anidulafungin and micafungin, while the two with identified mutations had MIC values above the ECV (non-WT). The rezafungin-NS *C. tropicalis* isolates had S654P and F650S mutations identified in the FKS1 HS1; these isolates were pan-R to other echinocandins. The four rezafungin-NS *C. parapsilosis* isolates were all WT in FKS1 HS regions (FKS2 was not tested). All four rezafungin-NS *C. parapsilosis* isolates were anidulafungin-NS, but S to caspofungin and micafungin.

## DISCUSSION

We examined the use of susceptibility testing results for other echinocandins as surrogate markers for rezafungin. Overall, there was high EA and CA for all agents in all *Candida* spp. when other echinocandins were used as a surrogate for rezafungin. VMEs were low when anidulafungin was used as a surrogate for rezafungin susceptibility but were higher when caspofungin or micafungin were used. Per CLSI guidelines, when a commercial system is used rather than the reference method, the categorical agreement should be ≥90%, VME < 3% of total resistant isolates, and ME <3% of total susceptible isolates (32). The FDA requires a more stringent VME of <1.5% for resistant isolates tested in commercial systems (28). There are no current guidelines on acceptable agreement for surrogate testing markers (24). Based on the recommended rates for commercial systems, if all *Candida* spp. are combined, anidulafungin would be the most appropriate surrogate marker for rezafungin susceptibility testing based on the VME of 2.9%, as caspofungin and micafungin overall had unacceptably high VME at 11.4% and 14.3%, respectively. Anidulafungin is also a favorable surrogate when looking at EA rates. All echinocandin agents performed at 100.0% using a cutoff of two doubling dilutions when compared to rezafungin. The two doubling dilution range is considered to be the range of reproducibility due to known variability in MIC testing. However, only anidulafungin displayed ≥90.0% EA among all *Candida* spp. when compared to rezafungin at one doubling dilution. Both micafungin and caspofungin had an unacceptably low EA rate at one doubling dilution among almost all organism categories. Only *C. glabrata* was ≥90.0% for caspofungin, and only *C. parapsilosis* was ≥90.0% for micafungin. Issues with caspofungin susceptibility testing are well established, resulting in high variability across laboratories, so the higher error rate with this agent is not surprising (33). However, the higher discrepancy rate between micafungin and rezafungin is remarkable, and we have no explanations for this observation. Rezafungin is most structurally similar to anidulafungin, and this may be why testing is most comparable between these two agents.

Species-specific differences were observed in the error rates for the different echinocandins. For *C. albicans*, all echinocandins performed well as surrogate markers for rezafungin susceptibility with zero VME. For anidulafungin, there were only two miE (<0.1%), and for caspofungin and micafungin, there were only two ME (<0.1%), so any of these agents would be appropriate to predict susceptibility to rezafungin for *C. albicans*. For *C. glabrata*, all agents also met benchmarks to be used as surrogates for rezafungin susceptibility testing. Anidulafungin had 0 VME, 10 (0.9%) ME, and 16 (1.4%) miE; caspofungin had 0 VME, 3 (0.3%) ME, and 9 (0.8%) miE; and micafungin had 0 VME, 6 (0.5%) ME, and 9 (0.8%) miE. All agents also worked well as surrogates for rezafungin when tested against *C. tropicalis* as there were no VME, ME, or miE for any agents. All agents also performed well when tested as surrogates for rezafungin against *C. krusei* with 0 VME, ME, or miE for anidulafungin or micafungin and 1 (0.5%) miE for caspofungin and no VME or ME.

VME were unacceptably high for both caspofungin and micafungin when compared to rezafungin for *C. parapsilosis* as all rezafungin-NS isolates tested susceptible to caspofungin (0.12–2 mg/L) and micafungin (1–2 mg/L) with breakpoints of ≤2 mg/L for both agents (VME four isolates, 100% of those tested). Only anidulafungin and micafungin could be compared with rezafungin for *C. dubliniensis* as there is no breakpoint or ECV defined for caspofungin against *C. dubliniensis*. Error rates were also unacceptably high when comparing anidulafungin or micafungin as a surrogate for rezafungin against *C. dubliniensis* (33.3% for both). These comparisons were between ECVs for anidulafungin and micafungin and a breakpoint for rezafungin. The high error rate may be due to lack of a breakpoint definition for other echinocandins, as a defined susceptible breakpoint is often a lower value than the NWT ECV breakpoint. Notably, the *C. dubliniensis* isolate leading to the VME was wild type in FKS1 HS1 and HS2 when the analysis was performed for resistance markers, whereas the two with agreement across categories both had the S645P substitution in FKS1 HS1.

The majority of isolates that tested NS to rezafungin had substitutions in the FKS1 and/or FKS2 HS regions that are known to lead to elevated echinocandin MIC values. Many of these isolates were similarly NS to other tested echinocandins. Unique among rezafungin-NS isolates, a single *C. glabrata* isolate appears to have undergone a 2,568 nucleotide recombination event between *fks1* and *fks2,* including the region harboring HS1. The resulting transfer of the S633P alteration may have produced a similar effect on echinocandin nonsusceptibility as mutation of the FKS1 HS1 S629P residue observed in other *C. glabrata,* as these residues occupy the same position in the respective FKS proteins. A similar recombination event has previously been described in a *C. glabrata* isolate from a patient treated with a prolonged course of micafungin resulting in an echinocandin-resistant isolate in Japan (34). It is interesting that we have identified a distinct occurrence of this complex mechanism of echinocandin resistance in *C. glabrata,* indicating that this organism may be prone to recombination between *fks* loci when placed under antimicrobial pressure. There were five isolates that did not have *fks* mutations but tested NS to rezafungin: one *C*. *dubliniensis* and four *C*. *parapsilosis*. These isolates warrant further study as their MICs fall into the susceptible or wild-type categories for the other echinocandins indicating that there may be resistance mechanisms to rezafungin outside of the known *fks* mutations leading to anidulafungin, caspofungin, and micafungin resistance particularly among *C. parapsilosis*.

### Conclusion

In this study, we evaluated anidulafungin, caspofungin, and micafungin as surrogate markers for rezafungin susceptibility testing against a collection of 5,720 IC isolates from the six most commonly identified *Candida* spp. We found that rezafungin susceptibility rates were high (≥97.6%) across species but that there are species- and echinocandin-specific differences in the performance of surrogate testing. The best surrogate was anidulafungin, which can be used to predict rezafungin S or NS in *C. albicans*, *C. glabrata*, *C. parapsilosis*, *C. tropicalis*, and *C. krusei* with low error rates and high essential and categorical agreement. Micafungin or caspofungin can be used as a surrogate marker for predicting rezafungin S or NS in *C. albicans*, *C. glabrata*, *C. tropicalis*, and *C. krusei*. No surrogate performs adequately to determine rezafungin susceptibility for *C. dubliniensis,* which may be due to lack of breakpoints for other echinocandins against this species and the use of ECVs for surrogacy. Rezafungin-NS isolates were assessed for alterations in FKS1 and FKS2 HS regions known to affect echinocandin susceptibility, and all but five rezafungin-NS isolates were identified with characteristic mutations in these regions. All of the rezafungin-NS *C. parapsilosis* isolates were nonsusceptible to anidulafungin but lacked mutations in FKS1, which may be due to the P660A polymorphism that has been described (35). The *C. dubliniensis* isolate that was NS to rezafungin may have unique genetic changes specific to rezafungin susceptibility as it was WT to other echinocandins; these isolates warrant further study.

## Data Availability

Whole-genome sequencing data are available under BioProject accession no. PRJNA1199623.

## SUPPLEMENTAL MATERIAL

Table S1 jcm.01129-24-s0001.docx Breakpoints and ECVs used in the study.

ASM does not own the copyrights to Supplemental Material that may be linked to, or accessed through, an article. The authors have granted ASM a non-exclusive, world-wide license to publish the Supplemental Material files. Please contact the corresponding author directly for reuse.
