# Supplementary material for: Anidulafungin is a useful surrogate marker for predicting in vitro susceptibility to rezafungin among five Candida species using CLSI methods and interpretive criteria
Source: J Clin Microbiol. 2025 Jan 22;63(2):e01129-24. doi: 10.1128/jcm.01129-24 (PMC11837505; doi:10.1128/jcm.01129-24)
Supplement: Table S1 — Breakpoints and ECVs used in the study. [file jcm.01129-24-s0001.docx]

| Supplemental Table 1: Defined breakpoints and ECV in mg/L for tested *Candida* spp. and echinocandins ^1,2^ | | | | | | | | | | | | | | | |
| --- | --- | --- | --- | --- | --- | --- | --- | --- | --- | --- | --- | --- | --- | --- | --- |
|  | Rezafungin | | | Anidulafungin | | | | Caspofungin | | | | Micafungin | | | |
| *Candida* spp. | S | I | R | S | I | R | WT | S | I | R | WT | S | I | R | WT |
| *C. albicans* | ≤0.25 |  |  | ≤0.25 | 0.5 | ≥1 |  | ≤0.25 | 0.5 | ≥1 |  | ≤0.25 | 0.5 | ≥1 |  |
| *C. glabrata* | ≤0.5 |  |  | ≤0.12 | 0.3 | ≥0.5 |  | ≤0.12 | 0.3 | ≥0.5 |  | ≤0.06 | 0.1 | ≥0.25 |  |
| *C. tropicalis* | ≤0.25 |  |  | ≤0.25 | 0.5 | ≥1 |  | ≤0.25 | 0.5 | ≥1 |  | ≤0.25 | 0.5 | ≥1 |  |
| *C. parapsilosis* | ≤2 |  |  | ≤2 | 4 | ≥8 |  | ≤2 | 4 | ≥8 |  | ≤2 | 4 | ≥8 |  |
| *C. krusei* | ≤0.25 |  |  | ≤0.25 | 0.5 | ≥1 |  | ≤0.25 | 0.5 | ≥1 |  | ≤0.25 | 0.5 | ≥1 |  |
| *C. dubliniensis* | ≤0.12 |  |  |  |  |  | ≤0.12 |  |  |  |  |  |  |  | ≤0.12 |

^1^ CLSI. 2022. M27 M44S Ed3. Performance standards for antifungal susceptibility testing of yeasts. Clinical and Laboratory Standards Institute, Wayne, PA

^2^ CLSI. 2022. M57S Ed4. Epidemiological cutoff values for antifungal susceptibility testing. Clinical and Laboratory Standards Institute, Wayne, PA.
